# Two-step PEG-based surface functionalization of gold nanoparticles enables specific SERS imaging of EGFR in an ovarian cancer cell model

**DOI:** 10.1016/j.mtbio.2026.103602

**Published:** 2026-08-27

**Authors:** Alessandro Stumpo, Ricardo Coelho, Mónica Núñez López, Lucy Kind, Jonathan De Roo, Francis Jacob, Sina Saxer

**Affiliations:** a University of Applied Sciences and Arts of Northwestern Switzerland (FHNW), Muttenz, Switzerland; b Ovarian Cancer Research, Department of Biomedicine, University Hospital Basel and University of Basel, Basel, Switzerland; c Department of Chemistry, University of Basel, Basel, Switzerland; d Swiss Nanoscience Institute, University of Basel, Basel, Switzerland

**Keywords:** SERS, Gold nanoparticles, PEG, Ovarian cancer, EGFR, CRISPR-Cas9 gene editing

## Abstract

Surface-Enhanced Raman Scattering (SERS)-based cancer imaging is of significant interest for histopathological examination, molecular analysis, and intraoperative guidance of tumor tissues due to its sensitive and specific detection of multiple cancer-associated biomarkers and stable signal over time. However, synthesis of SERS nanotags targeting cancer biomarkers is limited by the complexity in achieving a balance between Raman sensitivity, binding specificity, and nanoparticle stability. Here, we established a two-step PEG-based surface modification for 60 nm spherical gold nanoparticles to enable the SERS imaging of various ovarian cancer cell models expressing the epidermal growth factor receptor (EGFR). In contrast to conventional surface modification of gold-based SERS nanotags, our approach enables the simultaneous optimization of Raman activity, colloidal stability, and targeting specificity directly on gold nanoparticles. PEG-conjugated ⍺EGFR antibodies were successfully immobilized on 75.4 ± 4.6% of nanoparticles, with proof of accessible, active surface binding sites. Surface coverage with carboxyl-terminated poly(ethylene glycol)-1,4 benzenedithiol conjugate provided simultaneous SERS signal and steric stabilization, overcoming limitations associated with insufficient surface coverage of one or more functional groups. Colloidal stability during the two-step surface modification was maintained by the addition of surfactant Tween 80. Finally, SERS imaging demonstrated the specificity of our SERS nanotags toward EGFR-positive ovarian cancer cells (OVCAR5 and OVCAR8). Evaluation of *ΔEGFR*-OVCAR8 cells further confirmed that nanoparticle binding is primarily driven by specific antigen-antibody recognition, independent of cell line-specific effects. Overall, this work provides a promising strategy for effective SERS-based imaging of cancer, with potential for future clinical applications.

## Graphical abstract

**Figure:**
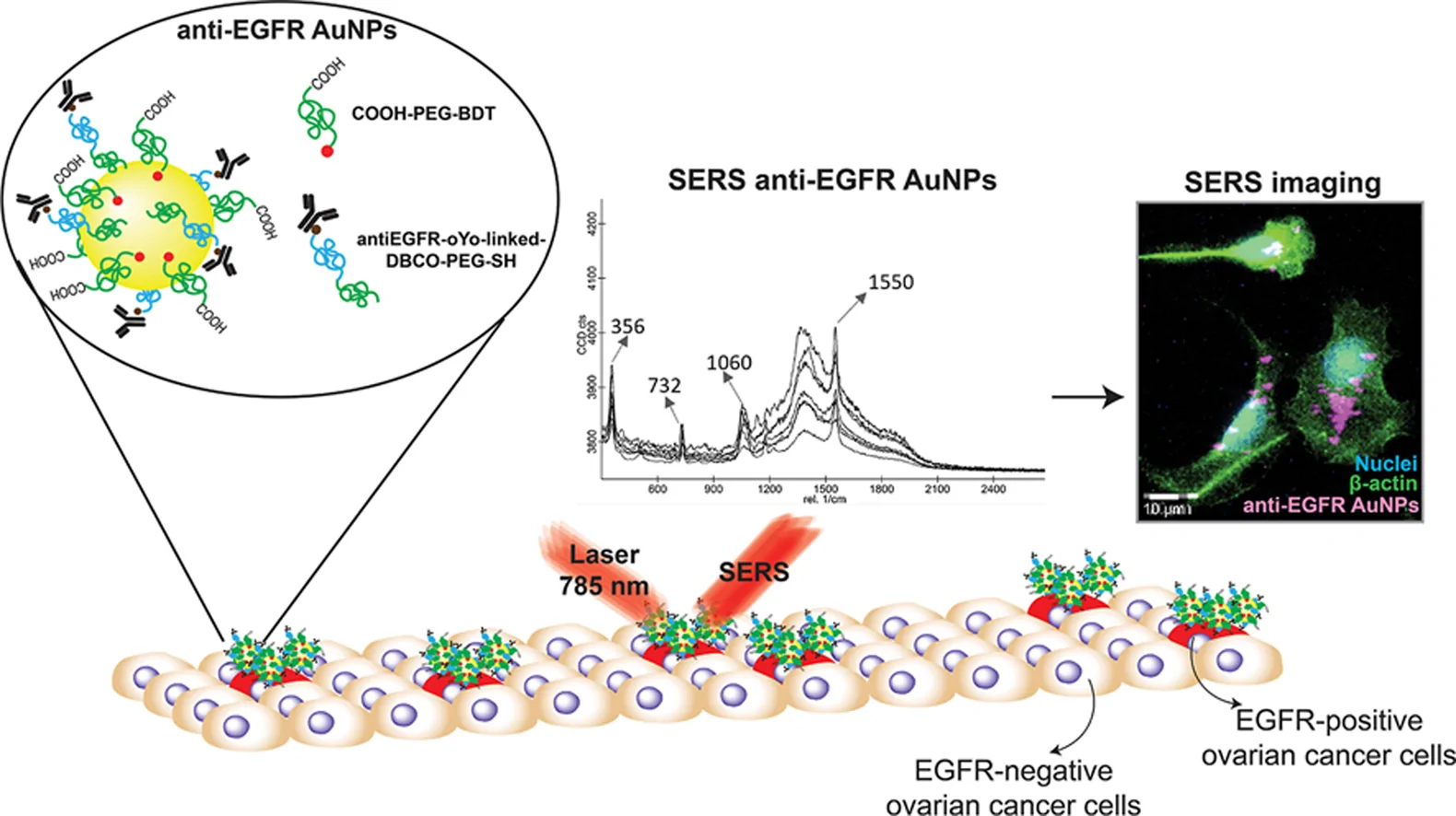

## 1 Introduction

Ovarian cancer (OC) is the gynaecological malignancy with the highest mortality [1], largely because its symptoms are silent, leading to diagnosis at an advanced stage in the majority of patients [2].

Despite advances in early-stage detection [3], histopathological examination of tumor tissue [[4], [5], [6]] remains essential for definitive diagnosis, staging, and treatment guidance [2,7]. However, conventional immunohistochemistry (IHC) is limited to single-marker detection, while multiplexed immunofluorescence (IF) is limited by spectral overlap of fluorophores [8,9]. Beyond diagnosis, cytoreductive surgery followed by chemotherapy remains the most common treatment for OC and a precise pre/intra-operative disease visualization of the disease is therefore essential, as patients with post-surgical residual tumors of less than 1 cm have a better prognosis [10]. Fluorescence-guided surgery is the current gold standard [11], enabling the *in situ* visualization of fluorescent agents that target tumor masses, such as 5-aminolevulinic acid (5-ALA) for glioblastoma and epithelial ovarian cancer [12]. However, cellular and tissue autofluorescence can lead to false positive results, and prolonged light exposure induces photobleaching of fluorescent agents and reduced imaging contrast.

Surface Enhanced Raman Scattering (SERS) represents a promising alternative to fluorescence-guided surgery and is of significant interest for molecular imaging in pathological contexts [[13], [14], [15]]. SERS-based cancer imaging relies on SERS active nanotags that selectively target cancer biomarkers. Due to its negligible photobleaching, narrow and fingerprint-specific Raman vibrational peaks, and exceptional sensitivity, SERS is ideally suited for highly sensitive, multiplexed and long-term stable labelling [[16], [17], [18]]. Moreover, the ability to use a single excitation laser for multiplexed analysis further highlights its clinical potential [19].

Gold spherical nanoparticles (AuNPs) are the most commonly used nanotags in biomedical fields due to their high biocompatibility compared with other plasmonic nanoparticles [20,21], and a relatively simple synthesis and strong surface functionalization via thiol-gold interaction. However, the surface functionalization and biomedical application of spherical AuNPs present several key challenges.

a) Sufficient surface coverage of all functional ligands. Functional SERS-active AuNPs require carefully balanced surface functionalization of a strong Raman marker (e.g., benzene-1,4-dithiol, BDT [22]), cell-specific targeting molecules (e.g., antibodies [23]), and stabilizers (e.g., polyethylene glycol, PEG [24,25]), to simultaneously ensure Raman sensitivity, binding specificity and nanoparticle stability [26]. Competitive binding and insufficient surface coverage of one or more functional groups results in compromised functionality. Achieving an adequate surface density of Raman reporters is particularly critical for spherical nanoparticles, as they exhibit lower SERS enhancement than other gold nanoparticle morphologies (e.g., nanostars, nanorods) or structures (e.g., nanoparticle assemblies) [27,28].
b) Reduced nanoparticle stability leads to agglomeration. Negatively charged nanoparticles, such as citrate-capped AuNPs, are stabilized via electrostatic surface charge repulsion. However, increasing ionic strength and variations in pH result in nanoparticle aggregation, unless additional steric stabilization is provided [29]. Cancer biomarker-targeting molecules such as antibodies require buffers with ionic strength that maintains their active biomolecular structure. The immobilization of antibodies on non-sterically stabilized AuNPs in saline buffers leads to nanoparticle aggregation and interparticle crosslinking driven by physical and chemical interaction (e.g., ionic and hydrophobic interaction, chemisorption via thiol groups) between the antibodies and the AuNPs surface [30,31]. Consequently, PEGylation is commonly required to ensure nanoparticle stability in physiological buffers. However, the high PEG surface density necessary for colloidal stability (∼1.13 chains/nm^2^) [32] restricts the available surface area for subsequent antibody immobilization, potentially resulting in low antibody density or even the absence of targeting molecules on the surface.
c) Gravitational sedimentation of single and aggregated nanoparticles. The sedimentation of AuNPs is heavily influenced by particle size and shape and is further accelerated by aggregation. This process must be carefully considered in *in vitro* studies and cell assays, as sedimenting nanoparticles deposit onto adherent cells, leading to nonspecific binding and cellular uptake [33], which can ultimately result in false positive SERS signals.
d) Nanoparticles adhesion to surfaces, affecting experimental reproducibility. Gold nanoparticles tend to adhere to surrounding surfaces such as glass or polymer. This phenomenon is further increased in saline buffers, such as phosphate buffered saline [34]. Such AuNPs adhesion becomes especially problematic during surface functionalization, which typically requires multiple washing steps (e.g., via centrifugation) to remove unbound molecules. During these steps, surface-bound AuNPs can be lost, thereby compromising experimental reproducibility.

One possibility to address all four major challenges is the use of SERS-active nanotags consisting of a gold core, a thin layer of Raman reporter adsorbed on the gold surface, and a protective silica coating [[35], [36], [37]]. While these nanotags provide sufficient Raman reporter loading and antibody surface density, along with enhanced stability [38], the synthesis of gold core-silica shell nanoparticles remains complex [[39], [40], [41]]. Common pitfalls include the formation of gold-free silica nanoparticles, silica-coated gold doublets, or incomplete and non-homogeneous silica shells [39].

Although PEGylation, thiol-gold chemistry, Raman reporter incorporation, and antibody-mediated targeting are established strategies for SERS nanotag development, their combination does not inherently overcome the challenges associated with biological implementation, including maintaining colloidal stability, generating sufficient SERS enhancement from spherical AuNPs, and preserving targeting specificity. Herein, we propose a well-defined SERS nanotag functionalization based on straightforward thiol-gold chemistry, enabling the incorporation of Raman-active molecules, PEG-based stabilizers, and targeting antibodies without the need for additional silica encapsulation or complex coating strategies. A two-step functionalization method was developed to synthesize PEG-based, 60 nm *anti*-EGFR gold nanoparticles, which were employed as nanotags for SERS imaging of epidermal growth factor receptor (EGFR)-expressing ovarian cancer cells *in vitro*.

## 2 Materials and methods

The following chemicals were purchased from Sigma-Aldrich, Inc. (St. Louis, MO, USA): 1,4-benzenedithiol (99%), Polysorbate 80 (Tween 80), 2,5-dihydroxybenzoic acid (DHB) (98%), 25% ammonia solution, 30% hydrogen peroxide solution, cysteamine (≥98%), 25% glutaraldehyde solution, Polysorbate 20 (>40%), ReadyBlue protein gel stain. The following chemicals were purchased from Biopharma PEG, Inc. (Watertown, MA, USA): maleimide-PEG-carboxylic acid (2 kDa) (≥95%), DBCO-PEG-SH (3.4 kDa) (≥90%). DMSO-*d*_6_ (99.8%) was purchased from Eurisotop (Saint-Aubin, France). 4% formaldehyde solution was purchased from Formafix AG (Hittnau, Switzerland). 60 nm gold nanoparticles (∼1.9E+10 particles/mL, ∼3.2 x 10^−2^ nM) and 40 nm gold nanoparticles (∼7.2E+10 particles/mL, ∼1.2 x 10^−1^ nM) were purchased from Sigma-Aldrich, Inc. And supplied pre-stabilized in 0.1 mM PBS, although the exact nature of the stabilizing agent was not disclosed. 150 nm gold nanoparticles (∼3.6E+9 particles/mL, 6.0 x 10^−3^ nM) were purchased from Sigma-Aldrich, Inc. stabilized in citrate buffer. Unless otherwise specified, Dulbecco's Phosphate-Buffered Saline (PBS), modified to exclude calcium chloride and magnesium chloride, sterile-filtered, and endotoxin-tested, was used throughout the study. Ultrapure water (resistivity 18.2 MΩ cm at 25 °C) was produced using an Arium® Pro Ultrapure Water System (Sartorius AG, Göttingen, Germany).

### 2.1 COOH-PEG-BDT conjugation

To synthesize COOH-PEG-BDT, 200 μL (0.002 mmol) of 1,4-benzenedithiol (BDT) dissolved in anhydrous DMSO (10 mM) and 200 μL (0.002 mmol) of maleimide-PEG-carboxylic acid aqueous solution (10 mM) were mixed in 1.6 mL of PBS buffer (pH 6.5-7.5) in a glass vial purged with argon gas and stirred with a magnetic stirrer at room temperature for 2 h. The reaction mixture was then aliquoted into 200 μL volumes, freeze-dried, and stored at −20 °C.

### 2.2 Conjugation of ⍺EGFR-oyo-linked-PEG-SH

To prepare the antibody conjugates, 6 μL (0.198 nmol) of DBCO-PEG-SH solution (33 μM) and 6 μL (0.0198 nmol) of oYo-Link® Azide solution (33 μM) (#AT3002, AlphaThera, Philadelphia, PA, USA) were mixed in 48 μL of PBS. The reaction was performed in a 0.6 mL tube and incubated at 750 rpm for 2 h at 37 °C (using Eppendorf Thermomixer compact; Eppendorf, Inc., Hamburg, Germany). Subsequentially, the mixture was transferred to 0.3 mL glass microvials, and 14.5 μL of recombinant ⍺EGFR antibody solution (1 mg/mL) (#10001-R021, Sino Biological, Inc., Beijing, China) was added. The solution was then exposed to UV light (366 nm) for 2 h on ice. Unreacted oYo-Link® Azide-DBCO-PEG-SH was removed using Amicon Ultra 0.5 mL centrifugal filters (MWCO: 50 kDa) by centrifugation at 14,000 ×g for 10 min. The purified antibody-conjugate solution was stored at 4 °C until use.

### 2.3 Surface functionalization of *anti*-EGFR AuNPs

In a 1.5 mL Eppendorf tube,1 μL of Tween 80 was added to 1 mL of AuNP solution (diameter 60 nm) and incubated for 4 h at 4 °C. The nanoparticles were then centrifuged at 4500 rpm for 10 min, and the pellet was resuspended in 100 μL of PBS with 0.01% Tween 80. Immediately, 10 μL of the ⍺EGFR-oYo-linked-PEG-SH conjugate was added and incubated for 2 min at room temperature. Then, 10 μL of the COOH-PEG-BDT conjugate was added and incubated for 10 min. The functionalized AuNPs were subsequently washed by centrifugation (3×, 4500 rpm, 15 min each) and resuspended in 0.5 mL PBS with 0.01% Tween 80. Samples were stored at 4 °C until further use.

### 2.4 High performance liquid chromatography (HPLC)

High-performance liquid chromatography (HPLC) analysis was performed using an Agilent® 1100 series system (Agilent Technologies, Inc., Santa Clara, CA, USA), equipped with a solvent degasser (G1379A), quaternary pump (G1311A), autosampler (G1313A), column compartment (G1316A), and diode array detector (G1315B). Samples (50 μL) were injected onto a Bio SEC-3 column (7.8 × 50 mm, 3 μm, 100 Å; Agilent) for analysis. The mobile phases consisted of ultrapure water (solvent C) and acetonitrile (solvent B), with a gradient elution profile as follows: 0 min, 80% C; 10 min, 80% C; 20 min, 30% C; 25-28 min, 80% C. The flow rate was maintained at 0.5 mL/min, and the column temperature was held at 37 °C. Detection was performed at a wavelength of 214.4 nm. Chromatographic data were processed using OpenLab CDS (Agilent Technologies, Inc., Santa Clara, CA, USA).

### 2.5 Nuclear Magnetic Resonance (NMR)

For ^1^H NMR characterization, 1 mg of sample was dissolved in 600 μL of DMSO-*d*_6_. Spectra were recorded using an ultra-shielded 400 MHz NMR spectrometer (Bruker BioSpin AG, Fällanden, Switzerland) with 5 mm NMR tubes. Raw data were processed and spectra visualized using Spectrus Processor software (ACD/Labs).

### 2.6 Matrix-Assisted Laser Desorption/Ionization Time-of-Flight (MALDI-TOF)

A 0.5 μL aliquot of the sample, diluted 1:1 in 2,5-dihydroxybenzoic acid (DHB) solution, was spotted onto an MSP 96 MALDI-TOF target plate (Bruker Scientific Instruments, Billerica, MA, USA) and allowed to air-dry at room temperature. Mass spectra were acquired using a microflex MALDI-TOF mass spectrometer (Bruker Scientific Instruments, Billerica, MA, USA) operated in positive linear mode. Data were collected over an *m*/*z* range of 800 - 21000 and processed with MATLAB (MathWorks, Natick, MA, USA).

### 2.7 NanoFlow cytometry

To quantify antibody immobilization, 100 μL of *anti*-EGFR AuNPs were stained with Alexa Fluor 647-conjugated anti-Rabbit IgG secondary antibody (#111-607-003, Jackson ImmunoResearch, Inc., West Grove, PA, USA) for 30 min on ice in the dark. Following incubation, nanoparticles were washed by centrifugation three times with PBS buffer to remove unbound antibodies before analysis. A 488 nm laser was used to determine nanoparticle size, while a 640 nm laser was used to detect Alexa Fluor 647 fluorescence and quantify the percentage of nanoparticles positive for surface-bound ⍺EGFR antibodies. For instrument alignment, focusing, and calibration, 0.25 μm fluorescent silica microspheres (NanoFCM, Inc., Xiamen, China) were used. 40 nm and 150 nm standard gold nanoparticles served as size references. Additionally, an Alexa Fluor 647-conjugated anti-Rabbit IgG secondary antibody by Cell Signaling Technology (#4414, Cell Signaling Technology, Inc., Danvers, MA, USA) was tested as divalent secondary antibody.

### 2.8 Quartz crystal microbalance with dissipation (QCM-D)

QCM-D sensor functionalization was performed by adapting the method described by M. Pohanka et al. [42] Experiments were conducted using gold coated QCM sensors (Biolin Scientific AB, Gothenburg, Sweden) with a fundamental resonance frequency of 5 MHz. Prior to functionalization, the sensors were cleaned in a 5:1:1 mixture of ultrapure water, ammonia (25%), and hydrogen peroxide (30%) at 75 °C. After rinsing with ultrapure water and drying, the sensors were activated by immersion in a 50 mg/mL cysteamine solution for 2 h at room temperature. Following three rinsing steps with ultrapure water, the sensors were incubated in a 5% glutaraldehyde solution for an additional 2 h. After another triple rinse with ultrapure water, 50 μL of EGF receptor (#10001-H08H, Sino Biological, Inc., Beijing, China) solution (0.25 mg/mL in PBS) was added and incubated overnight at 4 °C. For the negative control, a sensor was coated with BSA under identical conditions. The sensors were then rinsed three times with PBS and blocked with a 10 mg/mL gelatin solution for 1 h at room temperature. Before mounting them into the QCM-D measurement chambers, the sensors were rinsed a final three times with PBS. Measurements were carried out using a QSense Analyzer, and the data were processed using QSoft and analyzed with QTools software (Biolin Scientific AB, Gothenburg, Sweden).

### 2.9 Surface enhanced Raman spectroscopy (SERS) measurement

SERS spectra of dried *anti*-EGFR AuNPs deposited on a glass slide were recorded using a confocal Raman microscope (alpha300 R, WITec GmbH, Germany) equipped with a 785 nm excitation laser. The measurements were carried out with a laser power of 60 mW, an integration time of 0.5 s, 10 accumulations, and a 50× (NA = 0.55) objective lens. For reference measurements, a few milligrams of the powdered 1,4-benzenedithiol were similarly spotted onto a glass slide and analyzed under identical conditions. All spectral data were processed using WITec Five 5.3 software.

### 2.10 Dynamic light scattering (DLS) and electrophoretic light scattering (ELS)

A 1 mL aliquot of the gold nanoparticle solution suspended in PBS was transferred into a PMMA single-use cuvette (BRAND GMBH). DLS measurements were performed using a Zetasizer ZS instrument (Malvern Panalytical Ltd., U.K.). The measurements were conducted at a fixed backscattering angle of 173° and maintained at a constant temperature of 25 °C throughout the experiment. Refractive index (nD): 1.330; viscosity: 0.8872 mPa*s. The same instrument was used to determine the zeta potential by ELS. Prior to measurements, the nanoparticles were washed twice by centrifugation (10 min, 4500 rpm), the final pellet was resuspended in ultrapure water, and the suspension was transferred to a folded capillary zeta cell (DTS1070, Malvern Panalytical).

### 2.11 Ovarian cancer cell lines

Ovarian cancer cell lines OVCAR5 (RRID: CVCL_1628, Oncotest GmbH), OVCAR8 (RRID: CVCL_1629, Oncotest GmbH), TOV-112D (RRID: CVCL_3612, ATCC) and HEK293T (RRID: CVCL_0063, obtained through internal collaboration) were used in this study. All cancer cell lines were cultured in RPMI-1640 medium enriched with 10% FBS, 100 U/mL penicillin and 0.1 mg/mL streptomycin at 37 °C, 5% CO_2_. All the cell lines are STR profiled for cell line authentication and were regularly tested for the absence of Mycoplasma.

### 2.12 Immunofluorescence

Cells were initially trypsinized and seeded into μ-Slide 8 Well chambers (ibidi GmbH, Gräfelfing, Germany) at a density of 30,000 cells per well in a final volume of 300 μL and then cultured until a confluent monolayer was established. Upon reaching confluence, the culture medium was aspirated, and the cells were gently washed with PBS. Fixation was performed using a 4% formaldehyde solution for 10 min at room temperature, followed by three washes with PBS. Next, cells were permeabilized with 0.25% Triton X-100 for 5 min at room temperature, and subsequentially incubated for 1 h at room temperature with a blocking buffer containing 5% fetal bovine serum (FBS), 1% BSA, and 0.1% Triton X-100. After blocking, cells were incubated overnight at 4 °C with anti-human EGFR primary antibody (#10001-R021, Rabbit monoclonal, Sino Biological, Inc., Beijing, China) diluted 1:300 in antibody dilution buffer (1% BSA + 0.1% Triton X-100 in PBS). The following day, cells were washed three times with PBS containing 0.1% Polysorbate 20 (Tween 20) and then incubated with Alexa Fluor 647-conjugated anti-Rabbit IgG secondary antibody (#4414, Cell Signaling Technology, Inc., Danvers, MA, USA) diluted 1:1000 in antibody dilution buffer for 3 h, at room temperature in the dark. After incubation, the chambers were carefully removed, the wells were washed three times with PBS, and cell nuclei were stained with 100 μL ProLong™ Antifade Mountant with DAPI (#P36971, Thermo Fisher Scientific, Inc., Waltham, MA, USA). A 24 mm × 60 mm coverslip was placed over the stained sample, and the edges were sealed with nail polish to prevent drying. Samples were stored in the dark at 4 °C until imaging. Fluorescence images were acquired using FV3000 confocal laser scanning microscope (Olympus Global, Inc., Tokyo, Japan).

### 2.13 Molecular cloning of sgRNAs

Single guide RNAs (sgRNAs) targeting the epidermal growth factor receptor (EGFR, gene locus 7p11.2)-encoding gene were designed using Benchling (Benchling, Inc., San Francisco, CA, USA) and synthesized by Sigma-Aldrich, Inc. The sgRNA sequences were annealed, and ligated into the LRG2.1 plasmid (Addgene, #108098) using T4 DNA ligase (Qiagen GmbH, Hilden, Germany) to enable co-expression of sgRNA and EGFP. The ligation product was transformed into *E. coli Stbl3* competent cells, selected on ampicillin-containing plates, and purified using the ZR Plasmid Miniprep-Classic kit (Zymo Research, Irvine, CA, USA). Insertion of the sgRNA sequences was verified by Sanger DNA sequencing (Microsynth AG, Balgach, Switzerland) using the human U6 primer (5′- GAG GGC CTA TTT CCC ATG ATT -3′). SgRNAs with high-quality scores were selected for editing of the target gene (Supplementary Table 1).

### 2.14 Lentivirus production

Human Embryonic Kidney HEK293T cells were seeded in a T75 flask at ∼50% confluency 24 h before transient transfection. The cells were then co-transfected with 4 μg LRG2.1-sgRNA plasmid, 2 μg pMD2.G (Addgene, #12259), and 2 μg pCMVR8.74 (Addgene, #22036) using jetPEI (Polyplus, Illkirch-Graffenstaden, France) according to the manufacturer's protocol. After 24 h, the culture medium was replaced, and viral supernatant was collected at 48 h post-transfection. The supernatant was clarified by filtration through a 0.45 μm PVDF filter, aliquoted into cryotubes, and stored at −80 °C until use.

### 2.15 Generation of *AAVS1* and *EGFR*-edited cancer cells

Stable Cas9-expressing OVCAR8 cells [43] were transduced with a mixture of lentivirus encoding the *EGFR*-targeting sgRNAs and incubated for 24 h before medium replacement. Lentivirus carrying a sgRNA targeting the *AAVS1* locus served as a negative control. After an additional 48 h, cells were subcultured for three consecutive passages to eliminate residual free virus prior to downstream assays.

### 2.16 Flow cytometry

Cells were detached from culture flasks using Cell Dissociation Buffer, Enzyme-Free, Hanks’-based (Thermo Fisher Scientific, Inc., Waltham, MA, USA) to preserve surface markers. After resuspension in FACS buffer (PBS containing 1% w/v FBS), cells were stained with Alexa Fluor 647 conjugated to anti-human EGFR antibody (#352917 BioLegend, Inc., San Diego, CA, USA) for 20 min on ice in the dark. Samples were then washed three times, followed by staining with 0.1 μg/mL DAPI (BioLegend, Inc., San Diego, CA, USA) for 5 min under the same conditions. Prior to analysis, cells were washed three additional times and transferred to 96-well V-bottom plates. Flow cytometry gating was performed for each cell line individually to exclude debris, doublets, and dead (DAPI^+^) cells.

For *anti*-EGFR AuNP targeting, the same procedure was applied, except that 300’ 000 cells were first incubated with *anti*-EGFR AuNPs for 30 min on ice in the dark, following staining with Alexa Fluor 647-conjugated anti-rabbit IgG secondary antibody (#4414, Cell Signaling Technology, Danvers, MA, USA, 1:500 dilution) under identical conditions. All experiments were performed using a CytoFLEX flow cytometer (Beckman Coulter, Inc. Brea, CA, USA), and the obtained data were analyzed with FlowJo software.

### 2.17 SDS-PAGE & western blot

Cells were lysed in radioimmunoprecipitation assay (RIPA) buffer supplemented with protease inhibitors, and lysates were stored at −20 °C until use. Prior to analysis, protein concentration was determined using a bicinchoninic acid (BCA) assay (#23227 Thermo Fisher Scientific, Inc., Waltham, MA, USA). Samples were diluted in 4× loading buffer containing dithiothreitol (DTT) to a final concentration of 2 μg/μL, heated at 95 °C for 5 min, and separated on a 10% polyacrylamide gel. Proteins were transferred onto a methanol-activated polyvinylidene difluoride (PVDF) membrane by electroblotting. Membranes were blocked with TBST + 3% non-fat milk powder for 1 h at room temperature, then cut into three sections for incubation with recombinant ⍺EGFR antibody (#10001-R021, rabbit monoclonal, Sino Biological, Inc., Beijing, China), α/β-tubulin antibody (#2148, rabbit, Cell Signaling Technology, Inc., Danvers, MA, USA), or GFP antibody (#GTX113617, rabbit, GeneTex, Inc., Irvine, CA, USA) overnight at 4 °C. After three washes, membranes were incubated for 3 h at room temperature with mouse anti-rabbit IgG HRP-linked antibody (#7074, Cell Signaling Technology, Inc., Danvers, MA, USA). Protein bands were visualized using Thermo Scientific SuperSignal West Dura Extended Duration Substrate (#37071 Thermo Fisher Scientific, Inc., Waltham, MA, USA) and imaged accordingly.

For SDS-PAGE without transfer, samples were run on a 10% polyacrylamide gel and immediately stained with ReadyBlue protein gel stain overnight at 4 °C.

### 2.18 SERS imaging

Ovarian cancer cells were prepared for SERS imaging as follows. Cells were detached from culture flasks using Cell Dissociation Buffer, Enzyme-Free, Hanks’-based (Thermo Fisher Scientific, Inc., Waltham, MA, USA) to preserve surface markers. Cells were first incubated with *anti*-EGFR AuNPs for 30 min on ice in the dark, then stained with Alexa Fluor 647-conjugated anti-rabbit IgG secondary antibody (#4414, Cell Signaling Technology, Danvers, MA, USA) diluted 1:500 under identical conditions. Excess *anti*-EGFR AuNPs were removed by three centrifugation steps. Subsequentially, 20′000 cells were sorted using a CytoFLEX SRT Cell Sorter (Beckman Coulter, Inc. Brea, CA, USA) and allowed to adhere to an μ-Slide 8 Well cell culture slide (ibidi GmbH, Gräfelfing, Bayern, Germany) for 4 h. Cells were then fixed with 4% formaldehyde for 10 min at room temperature, followed by three washes with PBS. Next, the cells were permeabilized with 0.25% Triton X-100 for 5 min at room temperature, and nuclei were stained with 0.1 μg/mL DAPI (BioLegend, Inc., San Diego, CA, USA) for 5 min. After washing, the chambers were carefully removed. Fluoromount™ Aqueous mounting medium was applied to each sample, and a 24 mm × 60 mm coverslip was placed over. The edges were sealed with nail polish to prevent drying. Samples were stored in the dark at 4 °C until imaging. SERS imaging was acquired using a confocal Raman microscope (alpha300 R, WITec GmbH, Germany) equipped with a 785 nm excitation laser. The measurements were carried out with a laser power of 60 mW, an integration time of 0.5 s, and a 50× (NA = 0.55) objective lens. The instrument was equipped with an HXP 120 lamp (LEJ - Leistungselektronik JENA GmbH, Jena, Germany) for the simultaneous acquisition of fluorescence images (DAPI and FITC channels). All images were processed using WITec Five 5.3 software.

## 3 Results

### 3.1 Two-step PEG-based functionalization of gold nanoparticles for the development of stable and selective *anti*-EGFR SERS nanotags

**Fig. 1:**
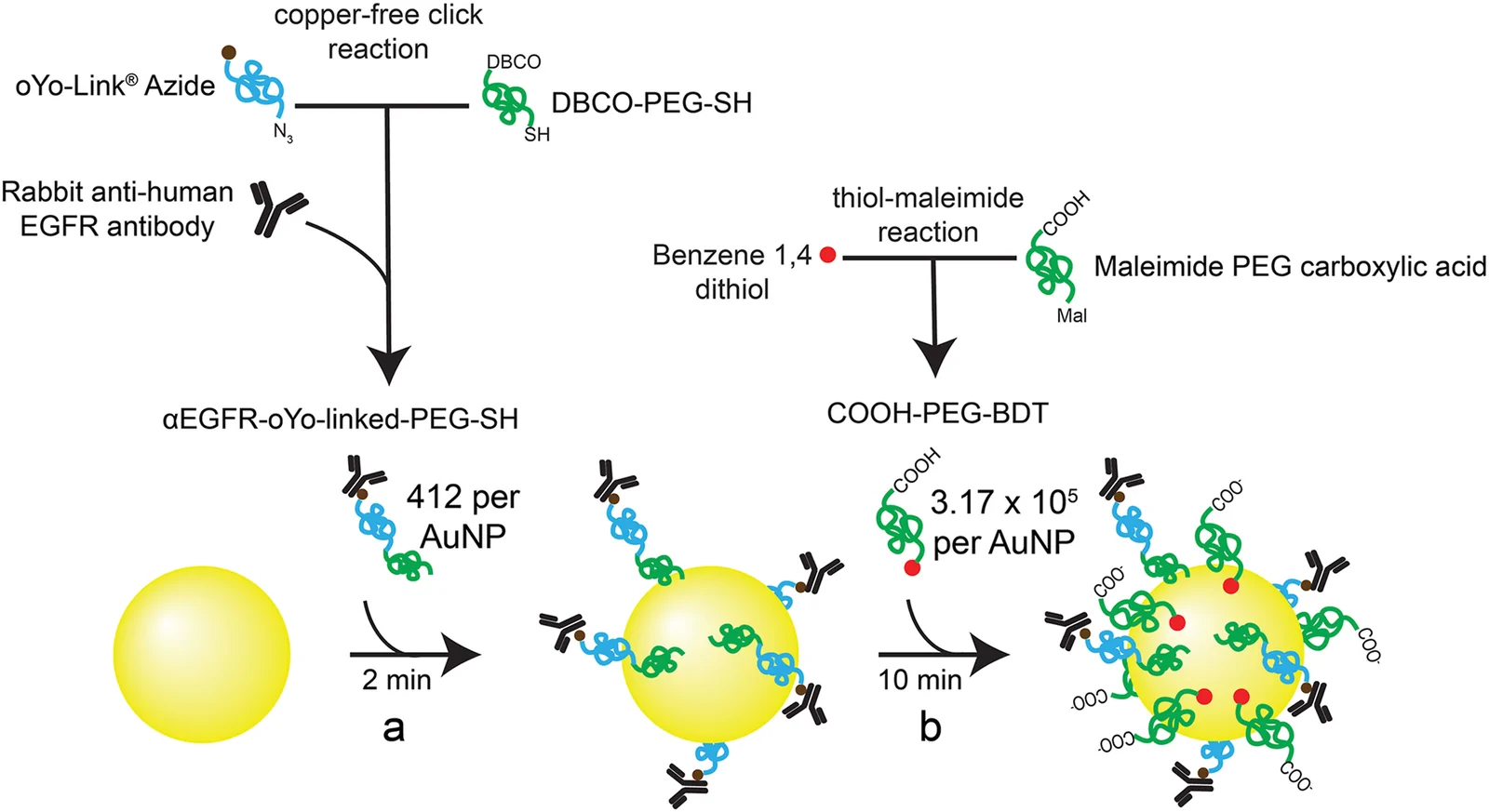
Schematic illustration of the two-step surface functionalization of 60 nm gold nanoparticles (AuNPs). Step a: anti-human EGFR antibodies, pre-conjugated to dibenzocyclooctyne-poly(ethylene)glycol thiol (DBCO-PEG-SH) via oYo-Link® Azide, are immobilized on the nanoparticle surface through gold-thiol interactions in PBS (0.01% Tween 80). **Step b**: Surface coverage with COOH-PEG-BDT, synthesized by conjugation of Benzene 1,4 dithiol (Raman reporter) to Maleimide PEG carboxylic acid, provides simultaneous steric stabilization and SERS functionality.

The functionalization of 60 nm AuNPs (Fig. S1) was done in a two-step reaction in PBS, chosen to preserve *anti*-EGFR antibody (⍺EGFR) activity. Because non-sterically stabilized AuNPs are not stable in buffer, the nanoparticles were temporarily stabilized prior to functionalization using the surfactant Polysorbate 80 (Tween 80, 0.01% v/v), providing colloidal stability. This concentration is well below those commonly used in immunoassays (0.05-0.1% v/v) and is therefore not expected to interfere with antibody activity. Additionally, the presence of Tween 80 in the washing buffer significantly reduced nonspecific nanoparticles surface adhesion, thereby enhancing the overall handling and reproducibility of the subsequent two-step functionalization procedure. First, ⍺EGFR-oYo-linked-PEG-SH conjugate was immobilized onto the AuNPs (412 conjugates/particle), followed by surface coverage with a 20-fold excess (20 x a theoretical PEG monolayer) of α-Maleimido-ω-carboxyPEG-benzene-1,4 dithiol (COOH-PEG-BDT) (Fig. 1). Although the commercial AuNPs were supplied with an undisclosed stabilizing agent, its weak interaction with the gold surface is suggested by the instability of the nanoparticles upon increasing ionic strength. We therefore expect efficient displacement by thiolated ligands during functionalization.

The two functional PEG-conjugates were synthesized and characterized independently prior to nanoparticles surface modification.

a) Conjugation of ⍺EGFR antibodies.

**Fig. 2:**
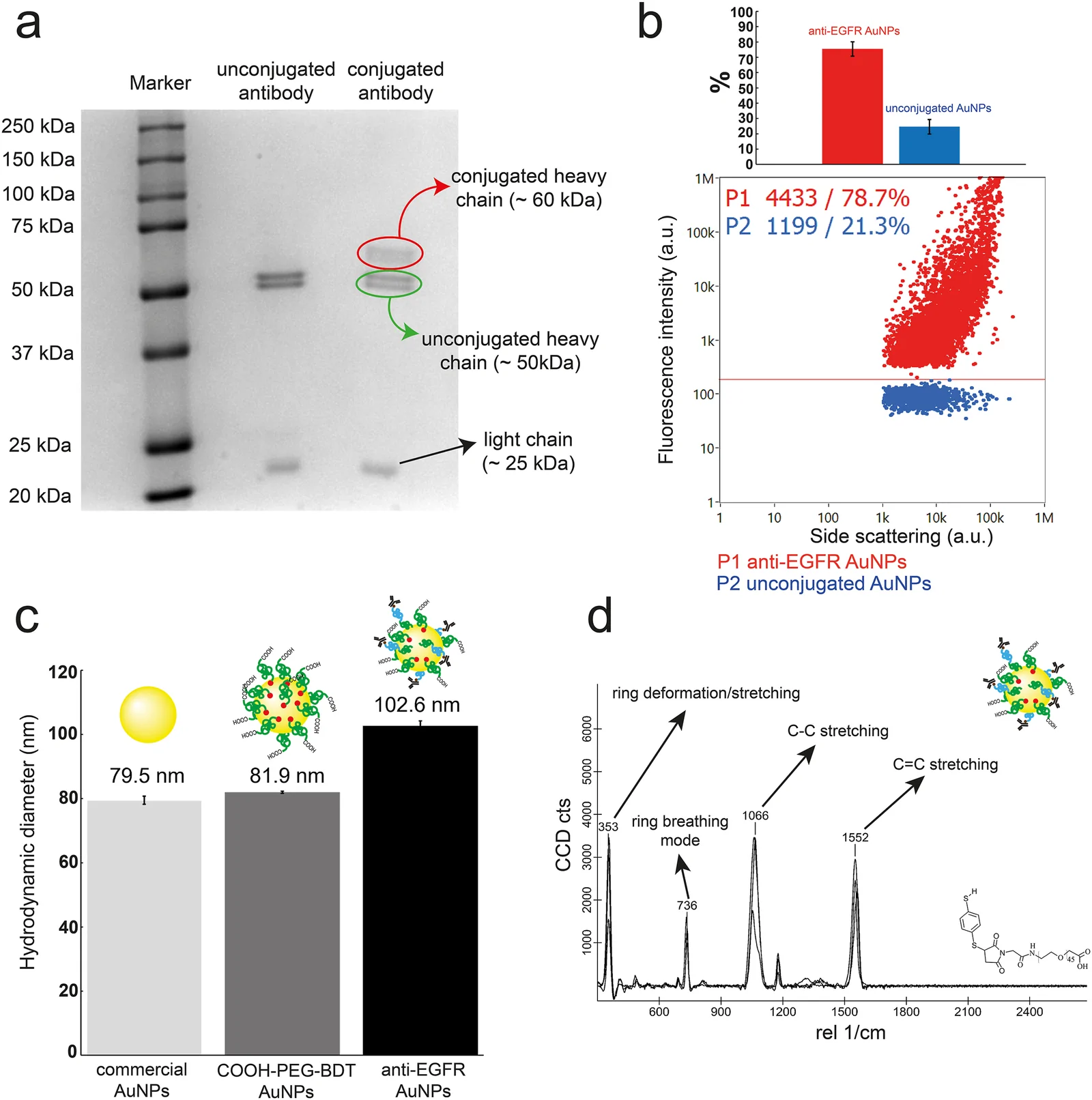
Characterization of the gold nanoparticle surface chemistry. a) Representative SDS-PAGE gel comparing the molecular weight of the unconjugated heavy chains of ⍺EGFR antibodies (∼50 kDa) with those of antibodies conjugated to oYo-Link® Azide-DBCO-PEG-SH (∼60 kDa). **b)** Representative Nano-Flow Cytometry graph showing the percentage of nanoparticles functionalized with ⍺EGFR antibodies (in red) after immobilization. Inset: mean percentages of *anti*-EGFR AuNPs (red) and unconjugated AuNPs (blue) from three independently functionalized samples. **c)** Dynamic light scattering (DLS) measurements highlighting differences in hydrodynamic diameter between non-functionalized AuNPs (commercial AuNPs), AuNPs stabilized without antibodies (COOH-PEG-BDT AuNPs) and fully functionalized AuNPs (*anti*-EGFR AuNPs). Error bars refer to three replicate measurements. **d)** Representative SERS spectra of *anti*-EGFR AuNPs (dry), highlighting the four main benzene 1,4 dithiol peaks (353 rel. 1/cm, 736 rel. 1/cm, 1066 rel. 1/cm, 1552 rel. 1/cm), with assignment to the corresponding vibrational modes.

Anti-human EGFR antibodies were attached to the DBCO-PEG-SH using Light-Activated Site-Specific Conjugation of Native IgGs (LASIC) [44] (Fig. S2). First, dibenzocyclooctyne-poly(ethylene)glycol thiol (DBCO-PEG-SH) was conjugated to oYo-Link® Azide via copper-free click reaction [45]. Complete conjugation was confirmed by high-performance liquid chromatography (HPLC) (Fig. S3), where the individual peaks corresponding to oYo-Link® Azide (7.3 min) and DBCO-PEG-SH (4.4 min) were no longer observed in the chromatogram of the conjugated product. A new peak at 5.6 min was observed, corresponding to the oYo-Link® Azide-DBCO-PEG-SH conjugate, with a secondary minor peak at 5.2 min likely corresponding to a reaction byproduct. The oYo-Link® Azide selectively recognizes the heavy chains of native IgGs and, upon UV light exposure, forms a covalent bond with the antibody. The oYo-Link® Azide-mediated conjugation of the antibody was confirmed by SDS-PAGE analysis (Fig. 2a), which revealed a distinct ∼10 kDa upward shift in the antibody heavy chain band, consistent with the molecular weight of the oYo-Link® Azide-DBCO-PEG-SH moiety (11.5 kDa). The residual band at 50 kDa corresponds to unmodified heavy chains, indicating incomplete conjugation. Extending UV exposure time up to 4 h did not further improve conjugation efficiency (Fig. S4). Consequently, unreacted oYo-Link® Azide-DBCO-PEG-SH was removed using Amicon® ultra centrifugal filters (MWCO: 50 kDa), while excess free antibody was removed by centrifugation after immobilization of ⍺EGFR-oYo-linked-PEG-SH on AuNPs, as only the thiol-functionalized antibody conjugates form stable interactions with the gold core.

The successful immobilization of antibodies to the AuNPs was assessed using Nano-Flow Cytometry after subsequent functionalization with COOH-PEG-BDT. On average, 75.4 ± 4.6% of the AuNPs displayed detectable ⍺EGFR antibodies on their surface, while 24.6 ± 4.6% showed no antibody immobilization (Fig. 2b, inset). It is important to note that this initial immobilization step of ⍺EGFR-oYo-linked-PEG-SH on AuNPs was performed in PBS (0.01% Tween 80), with steric stabilization only achieved after nanoparticles surface coverage with COOH-PEG-BDT. This resulted in a fraction of the nanoparticles aggregated (Fig. S5). Notably, only monovalent Fab fragments of the secondary antibody were compatible with nanoparticle staining, as conventional divalent secondary antibodies consistently induced irreversible particle aggregation (Fig. S7). Potential nonspecific adsorption was evaluated by incubating COOH-PEG-BDT AuNPs with secondary antibodies. The absence of fluorescence indicated negligible nonspecific binding. (Fig. S6).

To evaluate the targeting ability of our SERS-probe toward EGFR, Quartz Crystal Microbalance with Dissipation (QCM-D) measurements were conducted (Fig. S8). Recombinant EGFR and bovine serum albumin (BSA) were immobilized on separate gold-coated sensors via cysteamine and glutaraldehyde according to M. Pohanka et al. [42]. Anti-EGFR AuNPs caused a mass response of 30 ± 4.75 ng/cm^2^ (corresponding to ∼1.37 x 10^7^ particles/cm^2^) on the EGFR-coated sensor, whereas no significant response was detected on the BSA-coated reference sensor, confirming that the nanoparticles selectively bound to EGFR. An additional negative control was performed on the EGFR-coated sensor using COOH-PEG-BDT AuNPs. This experiment resulted in an unexpected negative mass shift, possibly indicating partial detachment of cysteamine from the sensor surface. Nevertheless, no mass increase was detected, suggesting that the COOH-PEG-BDT AuNPs did not bind to EGFR.

b) Conjugation of the Raman marker (BDT)

To achieve both strong SERS signal and colloidal stability, benzene-1,4-dithiol (BDT) was covalently linked to maleimide-PEG-carboxylic acid via a rapid thiol-maleimide reaction, forming a stable thiosuccinimide conjugate [46] (Fig. S9). The resulting COOH-PEG-BDT conjugate was then immobilized on the gold nanoparticle surface via gold-thiol interaction. Under physiological conditions, the negatively charged carboxylate group contributes an electrostatic component to the colloidal stability, on top of the steric stabilization provided by PEG (2 kDa), while the BDT core serves as Raman reporter. CH_3_O-PEG-BDT did not provide sufficient nanoparticle stabilization under the same conditions. The success of the thiol-maleimide conjugation was validated using Nuclear Magnetic Resonance (NMR) and Matrix-Assisted Laser Desorption/Ionization Time-of-Flight (MALDI-TOF) mass spectrometry. In the 1H NMR spectrum, the aromatic protons of maleimide at 7 ppm disappears completely in the reaction product, accompanied by the appearance of thiosuccinimide protons at 4 and 4.46 ppm, indicating complete conjugation (Fig. S10). MALDI-TOF mass spectrometry further confirmed successful conjugation by revealing a distinct molecular weight shift of 142.4 Da in the reacted maleimide, matching the theoretical mass of 1,4-BDT (142.24 Da) (Fig. S11). This is consistent with the formation of a 1:1 COOH-PEG-BDT product. Importantly, no signal was observed near 4000 Da, which corresponds to the formation of a COOH-PEG-BDT-COOH-PEG structure. The absence of such a peak suggests that both thiol groups of a single BDT molecule did not bind two different PEG chains under the experimental conditions.

Once COOH-PEG-BDT conjugate was immobilized on the *anti*-EGFR AuNPs surface, the characteristic SERS spectrum of the BDT moiety was detected, with four prominent peaks observed at 352 cm^−1^, 732 cm^−1^, 1061 cm^−1^, and 1550 cm^−1^ (Fig. 2d). A slight shift in peak positions compared to the BDT powdered reference (Fig. S12) was noted, due to the altered vibrational modes resulting from gold surface interactions and the local chemical environment.

Finally, changes in the hydrodynamic diameter and surface charge of both the *anti*-EGFR AuNPs and the COOH-PEG-BDT AuNPs were assessed using dynamic light scattering (DLS) and electrophoretic light scattering (ELS). An increase of approximately 20 nm was observed for the *anti*-EGFR AuNPs, which aligns with the expected size increment associated with successful surface conjugation of the antibody [47] (Fig. 2c). It was not possible to measure AuNPs functionalized only with ⍺EGFR-oYo-linked-PEG-SH due to particle instability at this intermediate stage of the functionalization process. In contrast, no significant difference in size was observed between the commercial AuNPs and the COOH-PEG-BDT AuNPs, likely because the purchased nanoparticles were supplied pre-stabilized in 0.1 mM PBS by the supplier using an undisclosed stabilizing agent. Surface charge measurements showed a clear change as the surface of the commercial AuNPs was functionalized, with a decrease from −59.03 ± 1.02 mV to −44.53 ± 1.55 mV for the *anti*-EGFR AuNPs. No significant difference was observed between nanoparticles with or without immobilized antibodies, as the COOH-PEG-BDT AuNPs showed a surface charge of −45.76 ± 0.66 mV (Fig. S13).

### 3.2 Development of an optimal cell model system for evaluating *anti*-EGFR AuNPs binding *in vitro*

**Fig. 3:**
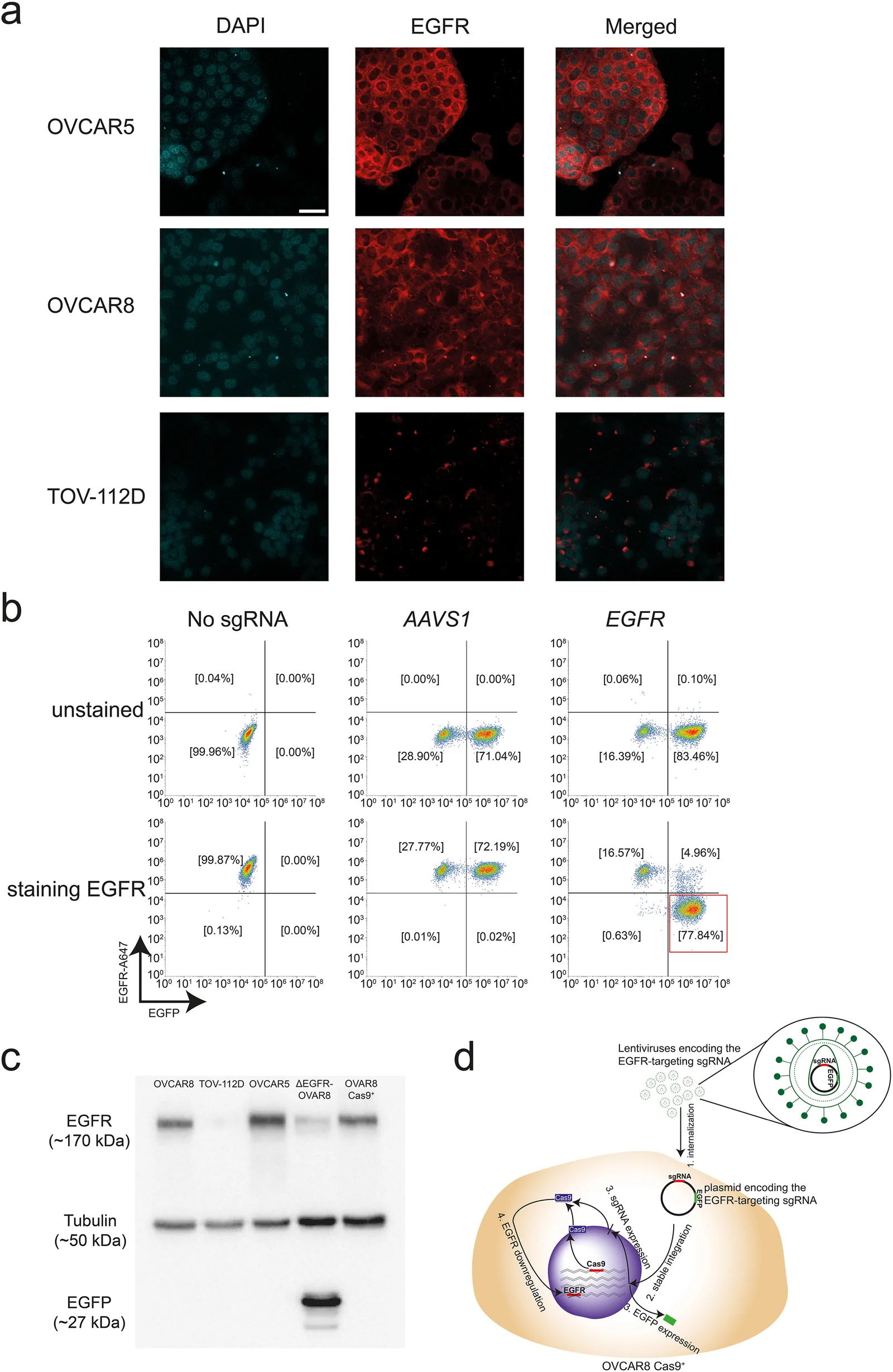
Characterization of EGFR expression and CRISPR-Cas9 mediated knockout across ovarian cancer cell lines. a) Representative confocal microscopy images of OVCAR8, OVCAR5, and TOV-112D. Nuclei were stained with DAPI, and EGFR with Alexa Fluor 647-labelled secondary antibody. Scale bar: 50 μm. **b)** Flow cytometry analysis of EGFR knockout in OVCAR8 Cas9^+^. Highlighted in a red box 77.84% of cells resulted EGFR-negative following transduction with lentiviral vectors carrying sgRNA targeting *EGFR*. Non-transduced cells (No sgRNA) and cells transduced with sgRNA targeting *AAVS1* were used as negative controls to confirm specificity and exclude off-target effects. The lentiviral construct also encoded EGFP to monitor transduction efficiency. **c)** Western blot comparing EGFR expression in OVCAR8, OVCAR5, TOV-112D, EGFR knockout OVCAR8 (*ΔEGFR*-OVCAR8), and Cas9-expressing OVCAR8 (OVCAR8 Cas9^+^). Tubulin was used as a constitutively expressed loading control. EGFP expression was detected only in transduced OVCAR8 cells (*ΔEGFR*-OVCAR8). **d)** Schematic illustration of lentiviral transduction of OVCAR8 Cas9^+^ cell with lentiviral vectors carrying plasmids encoding sgRNAs targeting *EGFR*.

The EGFR expression profiles of three ovarian cancer cell lines were characterized by confocal fluorescence microscopy. OVCAR5 and OVCAR8 cell lines exhibited positive EGFR expression, while TOV-112D is EGFR-negative (Fig. 3a). Nonspecific staining was observed on TOV-112D cells. Western blot analysis independently verified these findings, with EGFR protein bands detected in OVCAR5 and OVCAR8 cell lysates but absent in TOV-112D (Fig. 3c). Band intensity further indicated that OVCAR5 expresses approximately 1.5-fold higher levels of EGFR than OVCAR8.

To generate a more stringent negative control, EGFR expression in OVCAR8 cells was abolished by CRISPR-Cas9 genome editing, producing the *EGFR* knockout OVCAR8 line *ΔEGFR*-OVCAR8. Flow cytometry revealed that 77.84% of OVCAR8 cells transduced with sgRNAs targeting the *EGFR* gene locus showed loss of EGFR expression (Fig. 3b). Cells transduced with sgRNA targeting the *AAVS1* safe harbour locus, a genomic site whose disruption is not expected to affect cell viability or gene expression [48], retained unchanged EGFR expression, confirming editing specificity. Enhanced green fluorescent protein (EGFP) expression confirmed successful transduction of the sgRNA-encoding plasmids. A representative gating strategy is shown in Fig. S14. Western blot analysis further showed a marked reduction of the EGFR band in *ΔEGFR*-OVCAR8 (Fig. 3c), with a faint residual band consistent with the ∼20% of cells retaining EGFR expression.

### 3.3 SERS imaging of EGFR-positive OC cells

**Fig. 4:**
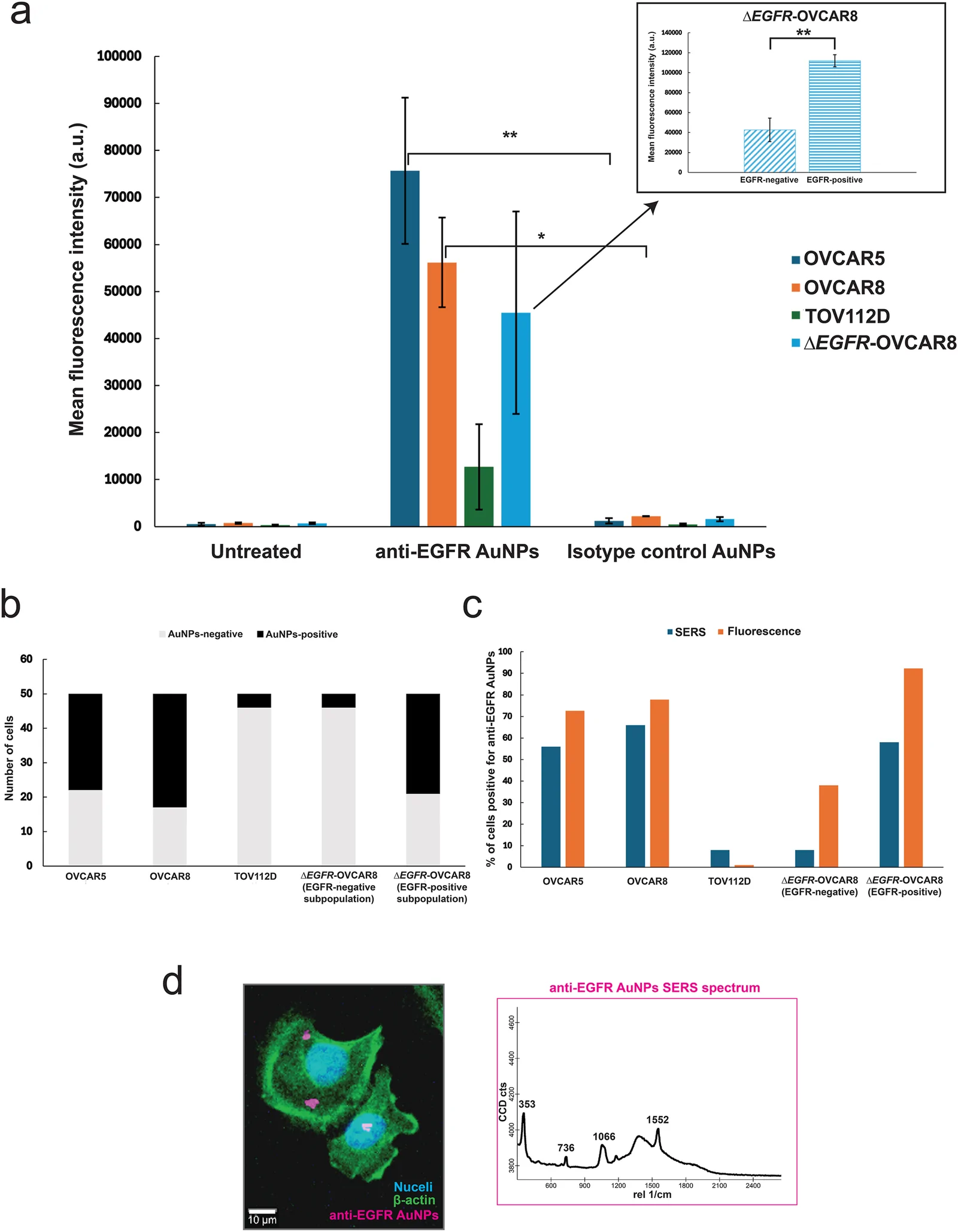
Selective binding and SERS imaging of *anti*-EGFR AuNPs in ovarian cancer cell lines of varying EGFR expression. a) Flow cytometry analysis assessing the binding specificity of *anti*-EGFR AuNPs toward EGFR-positive OC cells. Untreated cells and cells treated with isotype control IgG-functionalized AuNPs served as negative controls. The inset shows analysis of EGFR-positive and EGFR-negative subpopulations within *ΔEGFR*-OVCAR8 cells. Statistical significance was determined using an unpaired two-tailed Student's t-test and is indicated as follows: *p < 0.05, **p < 0.01 **b)** SERS measurement performed on 50 cells per cell line, with the number of cells positive (black) and negative (grey) for nanoparticle-derived SERS signal counted. **c)** Comparison of the percentage of ovarian cancer cells detected as positive for anti-EGFR AuNPs by SERS imaging and flow cytometry. **d)** Representative SERS imaging of two OVCAR5 cells with corresponding spectrum of cell-bound *anti*-EGFR AuNPs. Scale bar: 10 μm.

To validate the capability of *anti*-EGFR AuNPs to bind ovarian cancer cells expressing the EGFR and discriminate them from the EGFR-negative OC cells, flow cytometry analysis was initially performed (Fig. 4a). The *anti*-EGFR AuNPs selectively bound EGFR-positive OC cells (OVCAR5 and OVCAR8), while only minimal non-specific binding was observed for EGFR-negative cells (TOV-112D). The mean fluorescence intensity for TOV-112D was 5.9-fold and 4.4-fold lower than that of OVCAR5 and OVCAR8, respectively. Notably, analysis of the fluorescence intensity distributions of OVCAR8 and OVCAR5 revealed that *anti*-EGFR AuNPs-treated cells exhibited broader distributions compared to those treated with free ⍺EGFR antibodies (Fig. S15). This broadening is likely due to nanoparticle heterogeneity, as variations in antibody loading on the AuNPs surface result in differences in fluorescence intensity. To exclude potential variability coming from differences in nanoparticle-cell interactions between cell lines, *ΔEGFR*-OVCAR8 cells were included as an additional negative control. Given that ∼20% of the knockout cells still retained EGFR expression, this resulted in an overall mean fluorescence intensity that was 1.6-fold and 1.2-fold lower than that of OVCAR5 and OVCAR8, respectively. Further analysis of *ΔEGFR*-OVCAR8 showed that *anti*-EGFR AuNPs bound to the EGFR-positive subpopulation, with unspecific binding observed in the EGFR-negative subpopulation. The mean fluorescence intensity of the EGFR-negative subpopulation was 2.6-fold lower than that observed for the EGFR-positive subpopulation (Fig. 4a, inset). The fluorescence intensity distributions of the *ΔEGFR*-OVCAR8 cells treated with *anti*-EGFR AuNPs displayed a fluorescence profile similar to that obtained with ⍺EGFR antibodies (Fig. S15), with a small secondary fluorescence peak observed, corresponding to the residual subpopulation of cells that retained EGFR expression after genome editing. Finally, isotype control AuNPs functionalized with non-specific rabbit IgGs showed no significant interaction with either EGFR-positive or EGFR-negative cells, confirming that binding of *anti*-EGFR AuNPs is mainly driven by specific antibody-antigen interactions. The presence of isotype control IgG antibodies on the nanoparticle surface was confirmed using Nano-Flow Cytometry (Fig. S16).

Once the selectivity of the *anti*-EGFR AuNPs was confirmed, we assessed whether our nanoparticles can be used as SERS nanotags for discrimination between EGFR-positive and EGFR-negative OC cells by SERS imaging. For each OC cell line, 50 randomly selected cells were mapped by Raman microscopy, and the number of cells positive for AuNP-derived SERS signals was quantified (Fig. 4b). A cell was classified as SERS-positive if at least one pixel within the cell exhibited a Raman intensity above the selected threshold, defined as the mean background Raman intensity plus three times the standard deviations, for at least two characteristic BDT Raman peaks (352 cm^−1^, 732 cm^−1^, 1061 cm^−1^, and 1550 cm^−1^). SERS analysis revealed that the number of cells recorded as EGFR-positive by our SERS imaging system was 28, 33, and 29 out of 50 for OVCAR5, OVCAR8, and *ΔEGFR*-OVCAR8 (EGFR-positive subpopulation), respectively. In contrast, for EGFR-negative cells TOV-112D and *ΔEGFR*-OVCAR8 (EGFR-negative subpopulation) only 4 cells were erroneously recorded as EGFR-positive. These results confirm the high specificity of both our nanoparticles and the SERS imaging system for detecting EGFR-positive ovarian cancer cells. However, the percentage of EGFR-positive OC cells with bound nanoparticles detected by SERS imaging was consistently lower compared to that measured by flow cytometry (Fig. 4c). This observation suggests that some nanoparticle-bound cells may not be detected by SERS. To further investigate the relationship between EGFR expression, nanoparticle binding, and SERS response, the mean EGFR expression levels, *anti*-EGFR AuNP binding, and SERS brightness of OVCAR5 and OVCAR8 cells were compared (Fig. S17). As previously suggested by Western blot, OVCAR5 cells exhibited higher EGFR expression than OVCAR8 cells, which was accompanied by increased binding of *anti*-EGFR AuNPs as determined by flow cytometry, resulting in a 1.3-fold higher mean fluorescence intensity in OVCAR5 cells. However, despite this higher receptor expression and nanoparticle binding, the average SERS brightness was 1.6-fold higher in OVCAR8 cells than in OVCAR5. This observation indicates that SERS intensity does not scale directly with EGFR expression or the total number of nanoparticles bound to the cell surface.

Finally, Fig. 4d shows a representative SERS image of *anti*-EGFR AuNPs detected on two different OVCAR5 cells co-stained by fluorescence, together with the corresponding SERS spectrum. Nanoparticles bound to the cells appeared as distinct SERS-positive dots.

## 4 Discussion

This study demonstrates that the ability to discriminate between EGFR-positive and EGFR-negative ovarian cancer cells by SERS imaging is critically governed by the balance of the nanoparticle surface chemistry. Achieving this balance requires an optimized functionalization procedure that concurrently preserves colloidal stability, maximizes SERS signal intensity, and ensures binding specificity. The four challenges previously mentioned showed that colloidal stability and nanoparticle specificity can be rapidly compromised when surface chemistry is adapted.

The presence of Tween 80 throughout the entire functionalization process helped in maintaining the nanoparticles in suspension and preventing their adsorption onto surfaces. This approach also enabled the immobilization of PEG-conjugated ⍺EGFR antibodies on the nanoparticle surface in PBS while preserving their active biomolecular structure. Prior steric stabilization via thiol-based ligands resulted in reduced antibody loading on the nanoparticle surface, ultimately compromising targeting efficiency. Furthermore, separate sequential immobilization of Raman-active molecules and PEG-based stabilizers consistently resulted in either insufficient SERS enhancement or poor colloidal stability. In our strategy, the covalent conjugation of Benzene-1,4-dithiol to maleimide PEG carboxylic acid via thiol-maleimide chemistry proved to be essential. This bifunctional construct ensured the robust immobilization of the Raman-active molecule, providing highly detectable SERS signal while simultaneously stabilizing the nanoparticles in phosphate buffered saline. Notably, the SERS signal of the *anti*-EGFR AuNPs was recorded under dry conditions, as measurements performed in PBS did not show any signal, likely due to the relatively lower SERS enhancement of spherical nanoparticles compared to anisotropic structures.

Assuming an average antibody footprint of 25 nm^2^ [49], the number of IgGs required to form a single monolayer on a 60 nm gold nanoparticle is approximately 452. Using our approach, the theoretical number of immobilized antibodies per gold nanoparticle was calculated to be 412. However, any nonspecific adsorption of unconjugated antibodies to the AuNPs (likely driven by hydrophobic or electrostatic interactions) is expected to be displaced during the subsequent surface coverage with COOH-PEG-BDT. Therefore, the actual number of antibodies per nanoparticle is expected to be considerably lower. To assess the efficiency of the antibody immobilization, a validation method based on fluorescence detection of surface-bound antibodies using labelled secondary antibodies was established. Notably, the use of divalent secondary antibodies consistently induced nanoparticle crosslinking. This might be attributed to their ability to simultaneously bind primary antibodies on adjacent nanoparticles, effectively crosslinking them. Consequently, only monovalent antibody fragments could be used for NanoFCM staining. Through this optimized approach, *anti*-EGFR antibodies were detected on 75.4 ± 4.6% of AuNPs, which could bind to recombinant EGF receptor immobilized on QCM-D sensors, with an estimated surface density of ∼1.37 x 10^7^ particles/cm^2^.

*In vitro* tests designed to assess the binding specificity of our *anti*-EGFR SERS-nanotags needed to consider two important elements: a) the potential bias introduced by variability in nanoparticle interactions across different cell lines, some of which exhibit higher nanoparticle binding and uptake [50], and b) nanoparticle sedimentation onto adherent cells, which can lead to non-specific binding and uptake, resulting in false positive SERS artifacts. Nonspecific binding derived by gold nanoparticle sedimentation was minimized by incubation of cells with nanoparticles in suspension. However, removal of unbound nanoparticles by centrifugation alone was insufficient, as residual nanoparticles sedimentation still contributed to non-specific interactions. To overcome this limitation, cells were isolated via fluorescence-activated cell sorting prior to fixation and SERS imaging. Furthermore, to exclude cell line specific artifacts, CRISPR-Cas9 genome editing was used to knock out EGFR expression in 77.84% of OVCAR8 cells. The resulting *ΔEGFR*-OVCAR8 line served as a stringent, genetically matched internal negative control.

The final SERS imaging results highlighted two key aspects of our system: a) the robustness of our SERS imaging system across different cell lines, with EGFR-positive cell lines consistently resulting in ∼60% of cells recorded as positive, and EGFR-negative cell lines resulting in fewer than 10% of cells erroneously recorded as positive. This low false-positive rate confirms the high specificity of both our engineered surface chemistry and the confocal Raman configuration. b) in EGFR-positive OC cell lines, ∼40% of cells remain undetected by SERS imaging. This reduced sensitivity indicates that a subset of cells with lower nanoparticle density falls below the detection threshold of our current optical configuration. The short integration time (0.5 s) required for a rapid SERS cell imaging may be insufficient to detect weak or internalized SERS nanotags, which become visible only at longer laser exposure times. Consistently, some OVCAR8 cells initially recorded as negative exhibited a detectable SERS signal when the acquisition time was increased. This interpretation is further supported by the observation that the percentage of EGFR-positive cells recorded by SERS imaging is systematically lower than that measured by flow cytometry. Moreover, comparison of EGFR expression, *anti*-EGFR AuNP binding, and SERS brightness in OVCAR5 and OVCAR8 cells further demonstrates that SERS intensity does not directly correlate with receptor expression or the total number of nanoparticles bound to the cell surface. Although OVCAR5 cells exhibited higher EGFR expression and greater nanoparticle binding than OVCAR8 cells, the latter displayed a higher average SERS brightness. This observation reflects the fact that the SERS signal is strongly influenced by the local spatial organization of nanoparticles and the formation of plasmonic hotspots, where closely spaced nanoparticles generate substantially enhanced electromagnetic fields.

Notably, SERS imaging of *ΔEGFR*-OVCAR8 subpopulations strongly confirmed that cell-nanoparticle interaction is driven by specific antigen-antibody recognition, independent of cell line-specific effects. Our *anti*-EGFR SERS nanotags recognized 58% of cells in the EGFR-positive subpopulation, while only 8% of cells in the EGFR-negative subpopulation showed nonspecific nanoparticle binding.

Taken together, our results support the conclusion that effective SERS-based discrimination of cancer cells depends on a fragile balance between nanoparticle surface chemistry, colloidal stability, and targeting specificity. A reliable SERS imaging of cellular biomarkers is achieved only within a narrow operational window of SERS nanotag surface modification in which nanoparticle stabilization, Raman reporter immobilization, and antibody-mediated recognition are simultaneously optimized.

## 5 Conclusion

This study established a robust, two-step PEG-based surface modification strategy for spherical gold nanoparticles, achieving an optimal balance between colloidal stability, Raman sensitivity, and binding specificity for SERS imaging of ovarian cancer cells. Through systematic optimization of the functionalization procedure, we demonstrated effective, antigen-specific discrimination between EGFR-positive and EGFR-negative ovarian cancer cell models *in vitro*.

These findings provide a strong proof-of-concept for the development of tailored SERS nanotags targeting ovarian cancer biomarkers, offering promising potential for future clinical applications. Furthermore, the versatile surface engineering strategy presented here is broadly adaptable and can be extended to other cancer biomarkers, tumor types, and ultimately clinical samples.

## CRediT authorship contribution statement

**Alessandro Stumpo:** Writing – original draft, Visualization, Validation, Methodology, Investigation, Data curation. **Ricardo Coelho:** Writing – review & editing, Methodology, Investigation. **Mónica Núñez López:** Writing – review & editing, Methodology, Investigation. **Lucy Kind:** Writing – review & editing, Supervision. **Jonathan De Roo:** Writing – review & editing, Supervision. **Francis Jacob:** Writing – review & editing, Supervision, Funding acquisition, Conceptualization. **Sina Saxer:** Writing – review & editing, Supervision, Project administration, Funding acquisition, Data curation, Conceptualization.

## Declaration of competing interests

The authors declare that they have no known competing financial interests or personal relationships that could have appeared to influence the work reported in this paper.

## Data Availability

Data will be made available on request.
