# Supplementary material for: Two-step PEG-based surface functionalization of gold nanoparticles enables specific SERS imaging of EGFR in an ovarian cancer cell model
Source: Mater Today Bio. 2026 Aug 27;40:103602. doi: 10.1016/j.mtbio.2026.103602 (PMC13573746; doi:10.1016/j.mtbio.2026.103602)
Supplement: Supplementary file 1 [file mmc2.docx]

**Supplementary material**

**Fig. S1** Representative TEM image of 60 nm gold nanoparticles (left). HV: 50.0 kV; Magnification: 7000x. Nanoparticles size analysis was performed on 5 independent TEM images (right) using ImageJ software.

**Fig. S2** Schematic illustration of Light Activated Site-Specific Conjugation of Native IgGs (LASIC). Dibenzocyclooctyne-poly(ethylene)glycol thiol (DBCO-PEG-SH) is conjugated to oYo-Link^®️^ Azide via copper-free click reaction (37°C, 2 hours). The resulting product is then covalently linked to the heavy chains of anti-human EGFR IgGs upon UV light irradiation. Finally, the conjugated antibodies are immobilized on the gold nanoparticle surface via gold-thiol interaction.

**Fig. S3** Chromatogram of the conjugation of DBCO-PEG-SH to oYo-Link^®️^ Azide, validated by high-performance liquid chromatography (HPLC). The conjugated product exhibits a peak at a retention time of 5.6 minutes, while oYo-Link^®️^ Azide and DBCO-PEG-SH show retention times of 7.3 and 4.4 minutes, respectively. The absence of these peaks in the chromatogram of the conjugated product indicates complete conjugation. A minor peak at 5.2 min likely corresponds to a reaction byproduct.

**Fig. S4** Representative SDS-PAGE gel comparing the antibody conjugation efficiency following longer (4 h) and shorter (2 h) UV light exposure times. Band intensity analysis indicated that extended exposure did not significantly enhance conjugation efficiency, with ratios of conjugated to unconjugated heavy chain of 1.08 and 1.05 for 4 h and 2 h, respectively.

**Fig. S5** Size distribution of anti-EGFR AuNPs measured by Nano-Flow Cytometry. Some nanoparticles resulted aggregated, due to the high ionic strength conditions during the first functionalization step, prior to the steric stabilization.

**Fig. S6** Evaluation of non-specific adsorption of Alexa Fluor 647-labeled secondary antibody on COOH-PEG-BDT AuNPs by Nano-Flow Cytometry. No fluorescence signal was detected, suggesting that non-specific adsorption is negligible.

**Fig. S7** Comparison of anti-EGFR AuNPs staining with: (a) Alexa Fluor 647-labelled monovalent secondary antibody and (b) Alexa Fluor 647-labelled divalent secondary antibody. (b) resulted in a loss of solution colour, indicating gold nanoparticle aggregation.

**Fig. S8** Quartz Crystal Microbalance with Dissipation (QCM-D) measurements assessing the binding of anti-EGFR AuNPs to the EGF-receptor. Two gold sensors were coated with EGFR (black) and BSA (grey), respectively. Anti-EGFR AuNPs were then flowed over the sensors, and the resulting mass change was monitored (2). A significant mass increase was observed only for the EGFR coated sensor (30 ± 4.75 ng/cm2), corresponding to ~1.37 x 10⁷ particles/cm² bound to the surface. COOH-PEG-BDT AuNPs were flowed over the sensors (3) to evaluate non-specific binding, with no significant increase in mass registered. Negative mass changes likely indicate partial detachment of the cysteamine layer from the sensor surface.

**Fig. S9** Schematic illustration of the conjugation of maleimide-PEG-carboxylic acid with 1,4-benzenedithiol (BDT) via a rapid thiol-maleimide reaction.

**Fig. S10** Nuclear Magnetic Resonance spectra (^1^H-NMR, 400 MHz) of benzene-1,4-dithiol (BDT) (top), maleimide-PEG-carboxylic acid (middle), and COOH-PEG-BDT conjugate (bottom). The aromatic protons of maleimide-PEG-carboxylic acid (highlighted in red) at 7 ppm disappear completely in the COOH-PEG-BDT conjugate, with the thiosuccinimide protons appearing at 4 and 4.46 ppm. DMSO-d_6_ used as solvent contained 0.03% (v/v) of tetramethylsilane (TMS) as internal standard.

**Fig. S11** MALDI-TOF spectra of maleimide-PEG-carboxylic acid (blue) and COOH-PEG-BDT conjugate (red). A mass shift of 142.4 Da in COOH-PEG-BDT suggests successful conjugation of maleimide-PEG-carboxylic acid to benzene-1,4-dithiol (M.W.: 142.24 Da). No signal was detected at 4000 Da, excluding the formation of crosslinked molecules involving two maleimide-PEG-carboxylic acid molecules and one benzene-1,4-dithiol (COOH-PEG-BDT-COOH-PEG).

**Fig. S12** Raman spectrum of Benzene-1,4-dithiol (BDT), used as a reference for anti-EGFR AuNPs.

**Fig. S13** Zeta potential measurements highlighting differences in surface charge between non-functionalized AuNPs (commercial AuNPs), AuNPs stabilized without antibodies (COOH-PEG-BDT AuNPs), and fully functionalized AuNPs (anti-EGFR AuNPs). Error bars refer to three replicate measurements.

**Fig. S14** A representative gating strategy illustrating ΔEGFR-OVCAR8 being subgated to the level of EGFP+ and EGFR- cells.

**Fig. S15** Flow cytometry analysis of EGFR-positive (OVCAR5, OVCAR8) and EGFR-negative (TOV112D, ΔEGFR-OVCAR8) ovarian cancer cells treated with anti-EGFR AuNPs (blue), Alexa Fluor 647-labelled ⍺EGFR antibody (red), Isotype control AuNPs (yellow), and untreated. OVCAR5 and OVCAR8 treated with anti-EGFR AuNPs showed a clear shift in fluorescence intensity, suggesting nanoparticle binding, while no shift was observed for TOV112D. ΔEGFR-OVCAR8 showed a secondary peak corresponding to the subpopulation that retained EGFR expression. Untreated cells and cells treated with isotype control AuNPs served as negative controls, while cells treated with Alexa fluor 647-labelled anti-human EGFR antibody were used as a positive reference.

**Fig. S16** Representative Nano-Flow Cytometry graph showing the percentage of nanoparticles functionalized with rabbit IgGs (in red) after immobilization. Inset: mean percentages of Isotype control AuNPs (red) and unconjugated AuNPs (blue) from three independently functionalized samples.

**Fig. S17** Correlation between EGFR expression, anti-EGFR AuNP binding, and SERS brightness. The top graph shows higher EGFR expression in OVCAR5 than OVCAR8, determined by flow cytometry. The middle graph shows the binding of anti-EGFR AuNPs to OVCAR5 and OVCAR8 cells, quantified by flow cytometry after labeling the nanoparticle-bound antibodies with a fluorophore-conjugated secondary antibody. The bottom graph shows the SERS brightness relative to anti-EGFR AuNPs binding OVCAR5 and OVCAR8 cells.
